# Aetiology of Pleural Effusions in a Large Multicentre Cohort: Variation Between Outpatients and Inpatients

**DOI:** 10.1111/crj.13795

**Published:** 2024-10-09

**Authors:** Asfandyar Yousuf, Sophie Holland, Junyi Zhang, Cheryl Hardy, Madeline Charles‐Rudwick, Fredrik Vivian, Poppy Denniston, Nithin Thoppuram, Andrei Kisseljov, Rakesh K. Panchal, Eleanor K. Mishra

**Affiliations:** ^1^ Department of Respiratory Medicine Norfolk & Norwich University Hospital Norwich UK; ^2^ Department of Respiratory Medicine James Paget University Hospital Lowestoft UK; ^3^ Department of Respiratory Medicine Guys' and St Thomas' NHS Foundation Trust London UK; ^4^ Department of Respiratory Medicine, Glenfield Hospital University Hospitals of Leicester Leicester UK; ^5^ Faculty of Medicine University of East Anglia Norwich UK

**Keywords:** pleura, pleural effusions, pleural fluid, pleural neoplasm

## Abstract

**Introduction:**

This multi‐centre retrospective cohort study aimed to determine whether the cause of an undiagnosed pleural effusion differed depending on if a patient presented as an outpatient or inpatient.

**Methods:**

A total of 1080 adult patients (556 inpatients and 524 outpatients) presenting primarily with an undiagnosed pleural effusion from 1 January 2021 to 31 December 2022 from four UK hospitals were included.

**Results:**

We found malignant effusions were more common in outpatients compared to inpatients (48.3% vs. 36.0% *p* < 0.0001). Infection was common in inpatients but uncommon in outpatients (36.2% vs. 5.0% *p* < 0.0001). Other causes in all patients included heart and/or renal failure (13.1%) and non‐specific pleuritis (5.6%). No diagnosis was possible in 11.8% of patients referred.

**Conclusion:**

Investigative pathways should vary depending on whether patients present as an inpatient or outpatient.

## Graphical abstract

Our experience suggests that the underlying cause of a pleural effusion differs based on whether the patient presents as an emergency or if they present as an outpatient. In this large multi‐centre retrospective study, about half of outpatients had a malignant effusion with infection being rare, compared to about a third of inpatients having malignancy and infection being equally common.

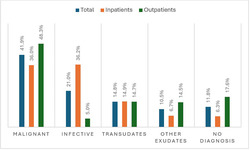

## 1 Introduction

Timely and accurate diagnosis of the underlying cause is vital for managing patients with a pleural effusion, as treatment approaches vary depending on the cause: prompt drainage and antibiotic treatment for pleural infection and biopsy and symptom control for those with malignant pleural effusions (MPE). The 2023 British Thoracic Society (BTS) pleural disease guidelines [1] propose a single pathway for investigation of patients who present with an undiagnosed pleural effusion.

Diagnosing and managing patients with pleural effusion in an outpatient specialist pleural unit [2] is becoming increasingly common in the United Kingdom [3], while inpatient diagnosis and management are typically reserved for those presenting as emergencies. This was reflected in the National Pleural Services Organisational Audit 2021, which identified that 48% of sites had a dedicated admission avoidance pathway for patients with pleural disease in the United Kingdom.

Previous studies have assessed the frequency of the different aetiology of pleural effusions. In a retrospective analysis of more than 3000 patients, Porcel et al. found that the leading causes of pleural effusions were malignancy (27%), heart failure (21%) and pneumonia (19%) [4]. In an epidemiological study of alive and dead patients, Morel et al. reported the most common aetiology to be heart failure (46%), malignancy (22%), parapneumonic effusions (17%) and pulmonary emboli (5.6%) [5].

Our clinical experience is that the underlying cause of a pleural effusion differs based on whether the patient presents as an emergency and is investigated as an inpatient or if they present as an outpatient and are investigated in pleural clinic. The aim of this study was to determine whether there are significant differences in the cause of pleural effusions based on mode of presentation.

## 2 Methods

We conducted a multi‐centre retrospective cohort study across four hospitals in the United Kingdom. The four centres consisted of three tertiary care hospitals (Norfolk & Norwich University Hospital [NNUH], Glenfield Hospital, University Hospitals of Leicester [GH] and Guy's & St. Thomas' NHS Foundation Trust [GST]) and one district general hospital (James Paget University Hospital [JPUH]). All hospitals had a dedicated pleural service and access to pleural biopsy (image guided or local anaesthetic thoracoscopy).

We included adult patients (over 18 years) with a primary presenting problem of an undiagnosed pleural effusion who underwent a diagnostic procedure (pleural aspiration, medical thoracoscopy or pleural biopsy) from 1 January 2021 to 31 December 2022. Diagnostic criteria were based on the 2023 BTS pleural disease guidelines [1]. We defined outpatients as patients who presented to pleural clinics for their first procedure. Inpatients were those who had their first diagnostic procedure as an inpatient. Patients with an incidental finding of a pleural effusion and patients who developed an effusion during their inpatient stay were excluded. Patients were identified by reviewing local pleural procedure logbooks and from clinical coding. The study protocol was reviewed by the ethics committee at NNUH. As the study involved retrospective data collection with no impact on patient management, formal ethical approval was not required. Local clinical governance departments gave approval prior to sharing anonymised data between sites. Data were collected and compiled on a standardised Excel 365 (Microsoft) spreadsheet. Graph‐pad Prism version 10 (Dotmatics) was used for data analysis, specifically employing Fisher's exact test. Details of how diagnoses were categorised are summarised in Table 1.

**TABLE 1:** Diagnostic criteria.

Malignant pleural effusion	Either one of: cytological or histological evidence of malignancy in pleural fluid or pleural biopsy; multidisciplinary team agreement that the effusion is malignant; recurring effusion in a patient with disease progression due to a known malignancy which was managed by the treating clinician as malignant pleural effusion.
Infective	
Pleural infection	Either one of: pus in the pleural space; clinical signs of infection associated with low pleural fluid pH/glucose; clinical signs of infection with a positive pleural fluid culture.
Simple parapneumonic effusion	Parapneumonic effusions that appeared simple (anechoic with no septations) on ultrasound in the absence of features of pleural infection as described above.
Tuberculosis	Either one of: isolation of pleural TB on cytology or histology; lymphocytic unilateral pleural effusion in the presence of underlying TB managed by the treating clinician as TB pleuritis.
Transudates	
Heart and/or renal failure	Underlying diagnosis of heart and/or renal failure with either one of: biochemical confirmation of a transudative effusion by using Light's criteria or serum‐effusion albumin gradient greater than 1.2 g/dL; strong clinical suspicion such as resolution of effusion after treatment of underlying cause.
Hepatic hydrothorax	Underlying diagnosis of liver failure with either one of: biochemical confirmation of a transudative effusion by using Light's criteria or serum‐effusion albumin gradient greater than 1.2 g/dL; strong clinical suspicion such as resolution of effusion after treatment of underlying cause.
Other exudates	
Non‐specific pleuritis	Histological confirmation by pleural biopsy
Pulmonary embolism	Pleural effusion on the side of pulmonary embolism in the absence of any other obvious cause.
Haemothorax	Either one of: presence of frank blood on pleural aspiration; pleural fluid haematocrit more than 50%.

## 3 Results

**FIGURE 1:**
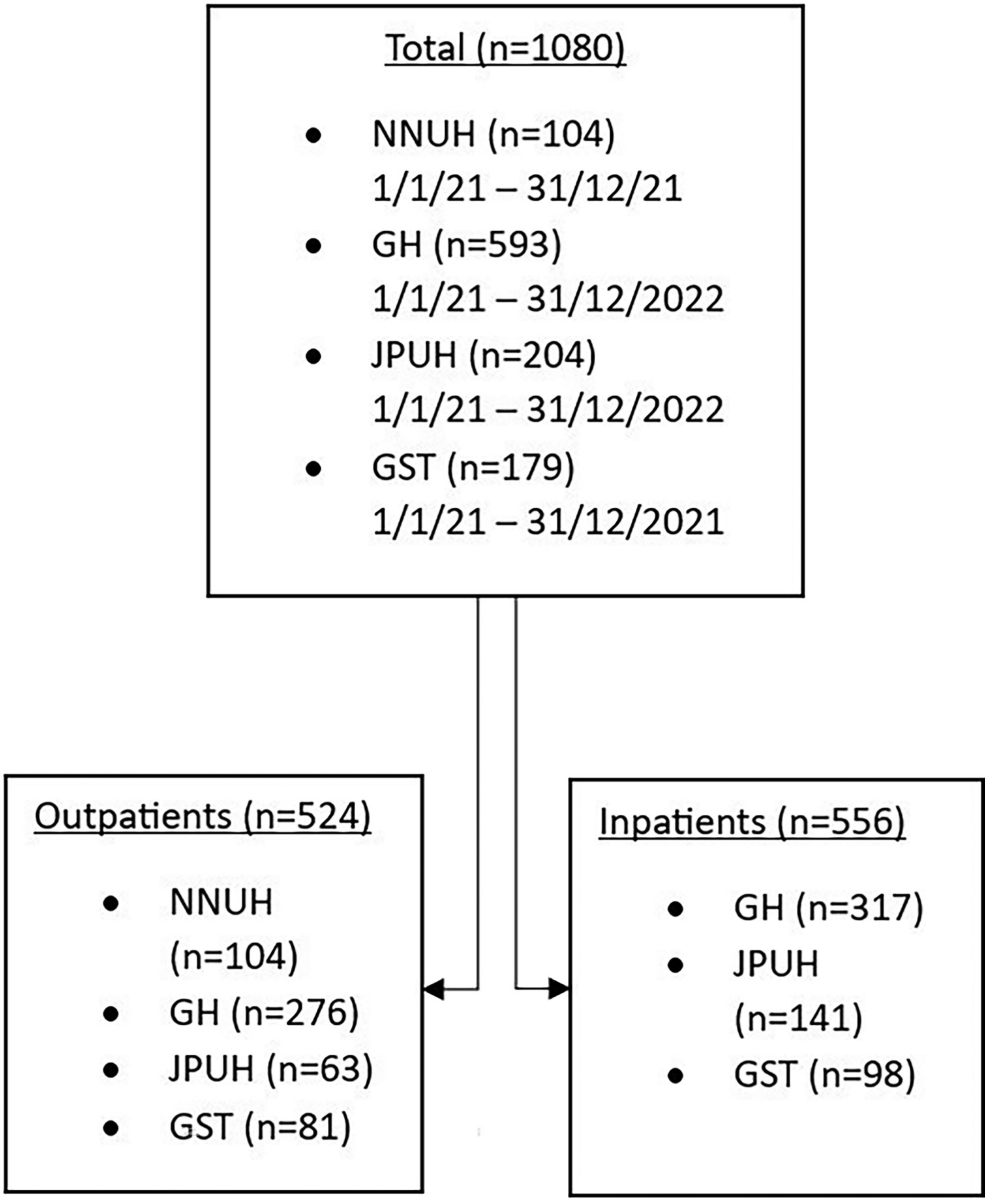
Patient distribution according to sites.

A total of 1080 patients were identified across four sites, 556 were inpatients and 524 were outpatients. Complete data for 2 years were available from two centres (GH and JPUH). Two centres provided data for 1 year only (NNUH and GST), and inpatient data were not available from one centre (NNUH). Patient distribution according to sites is described in Figure 1.

Of outpatients, 38.5% were female compared to 43.7% of inpatients. Mean age was 71.2 years (standard deviation [SD] 13.4) in outpatients and 68.8 years (SD 15.8) in inpatients. The majority of patients (72.5% of outpatients and 63.5% of inpatients) were 65 years of age or older.

Overall, the commonest causes of effusion for all patients were malignancy (41.9%), infective (21%) and heart and/or renal failure (13.1%). The commonest malignant diagnoses were lung cancer (32.5%), mesothelioma (17.4%), breast cancer (16.6%) and ovarian cancer (9.7%).

**FIGURE 2:**
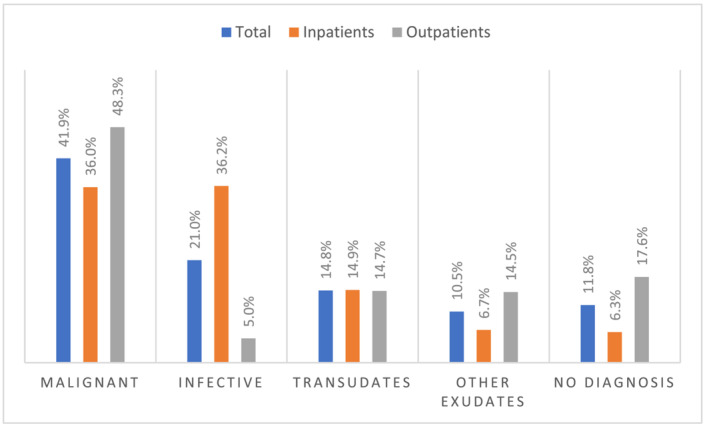
Distribution of diagnostic groups in the total population, outpatients and inpatients.

Malignant effusions were more common in outpatients compared to inpatients (48.3% vs. 36.0% Fischer exact test *p* < 0.0001). Infective effusions were more common in inpatients (36.2% vs. 5.0% Fischer exact test *p* < 0.0001). A significant minority (11.8%) of pleural effusions remained undiagnosed, this was a more common outcome in outpatients compared to inpatients (17.6% vs. 6.3% Fischer exact test *p* < 0.0001). Patient frailty was the most common reason for no diagnosis (40.2% in outpatients and 42.3% in inpatients). Figure 2 illustrates distribution of diagnostic groups while further details of diagnoses can be found in Table 2.

**TABLE 2:** Distribution of diagnoses categorised by outpatients and inpatients.

	Outpatient	Inpatient
Mean age in years (standard deviation)	71.2 (13.4)	68.8 (15.8)
Female sex (%)	38.5	43.7

Diagnosis	*n* (%)	*n* (%)	Fischer's exact test (*p*)
Malignant^a^	253 (48.3)	200 (36.0)	< 0.0001
Mesothelioma	52 (20.5)	27 (13.5)	
Lung cancer	74 (29.3)	73 (36.5)	
Haematological malignancy	18 (7.1)	17 (8.5)	
Ovarian cancer	19 (7.5)	25 (12.5)	
Breast cancer	46 (18.2)	29 (14.5)	
Gastrointestinal cancer	26 (10.3)	14 (7.0)	
Other^b^	18 (7.1)	16 (2.9)	
Infective^a^	26 (5.0)	201 (36.2)	< 0.0001
Pleural infection	9 (34.6)	125 (62.1)	
Simple parapneumonic effusion	14 (53.8)	64 (31.8)	
Tuberculosis	3 (11.5)	12 (6.0)	
Transudates			
Heart and/or renal failure	70 (13.4)	71 (12.8)	0.79
Hepatic hydrothorax	7 (1.3)	12 (2.2)	0.36
Other exudates^a^	76 (14.5)	37 (6.7)	
Non‐specific pleuritis	55 (72.4)	5 (13.5)	
Pulmonary embolism	1 (1.3)	10 (27.0)	
Haemothorax	6 (7.9)	6 (16.2))	
Postsurgical	3 (3.9)	5 (13.5)	
Autoimmune pleuritis	2 (2.6)	2 (5.4)	
Reactive to intra‐abdominal pathology	2 (2.6)	0	
Chylothorax^c^	2 (2.6)	0	
Drug related	1 (1.3)	1 (2.7)	
Miscellaneous^d^	4 (5.3)	8 (21.6)	
No diagnosis^a^	92 (17.6)	35 (6.3)	< 0.0001
Frailty	37 (40.2)	15 (42.9)	
Effusion too small for safe aspiration	21 (22.8)	8 (22.9)	
Patient choice for observation	8 (8.7)	3 (8.6)	
Patient died before diagnosis	7 (7.6)	3 (8.6)	
Spontaneous resolution	19 (20.7)	6 (17.1)	

^a^ Subcategories for these categories have percentages expressed as a proportion of the categories rather than the total population.

^b^ Other malignant diagnoses included prostate cancer, melanoma, renal cell cancer, oropharyngeal cancer, endometrial cancer, sarcoma, thyroid cancer, bladder cancer and unknown primary.

^c^ One patient with chylothorax secondary to liver cirrhosis and cause uncertain in second patient.

^d^ One each of Erdheim Chester disease, yellow nail syndrome, IgG4 disease, effusion post CT biopsy, covid‐related hydropneumothorax, effusion with vaccine‐induced thrombocytopaenic purpura, effusion post transcatheter aortic valve implantation, Dressler syndrome, CAPD diasylate fluid causing effusion and reactive effusion secondary to infective endocarditis.

## 4 Discussion

This multicentre retrospective cohort study demonstrates that the aetiology of pleural effusions varies between inpatients and outpatients. Around half of patients presenting as outpatients have malignancy with infection being a rare cause in this group, whereas about a third of inpatients have infection with malignancy being equally common.

Another finding of note was that around 10% had an effusion secondary to heart and/or renal failure, which is lower than other series [4, 5]. Pulmonary embolism and autoimmune pleuritis were uncommon, despite the BTS pleural disease guidelines listing these as common causes of exudative effusions. Non‐specific pleuritis was more common.

The strengths of these data are that they come from a large multi‐centre cohort and are therefore likely to be reflective of real‐world experience. Limitations are that criteria for admitting patients varied across sites depending on local expertise and experience. In addition, there is the possibility of selection bias due to heterogeneity of coding systems available in different centres.

In summary, amongst patients presenting with a primary problem of a pleural effusion, about half of outpatients have MPE with infection being rare, compared to about a third of inpatients having MPE and infection being equally common. Given this wide variation, we propose that the pathway for investigation of pleural effusions should vary based on presentation of the patient. In patients presenting to outpatient pleural clinics, an upfront CT scan and early biopsy are key. In contrast, the key challenge for inpatients is to distinguish between patients with infection and malignancy.

## Author Contributions

A.Y. was responsible for the study design, data acquisition and analysis, drafted and revised the manuscript, provided final approval of manuscript and agreed accountability for all aspects. S.H. provided substantial contribution to data acquisition, drafted and revised the manuscript, provided final approval of manuscript and agreed accountability for all aspects. J.Z. provided substantial contribution to data acquisition, drafted and revised the manuscript, provided final approval of manuscript and agreed accountability for all aspects. C.H. provided substantial contribution to data acquisition, revised the manuscript, provided final approval of manuscript and agreed accountability for all aspects. M.C.R. provided substantial contribution to data acquisition, revised the manuscript, provided final approval of manuscript and agreed accountability for all aspects. F.V. provided substantial contribution to data acquisition, revised the manuscript, provided final approval of manuscript and agreed accountability for all aspects. P.D. provided substantial contribution to data acquisition, revised the manuscript, provided final approval of manuscript and agreed accountability for all aspects. N.T. provided substantial contribution to data acquisition, revised the manuscript, provided final approval of manuscript and agreed accountability for all aspects. A.K. provided substantial contribution to data acquisition, revised the manuscript, provided final approval of manuscript and agreed accountability for all aspects. R.K.P. was responsible for the study design, revised the manuscript, provided final approval of manuscript and agreed accountability for all aspects. E.K.M. was responsible for the study conception and design, revised the manuscript, provided final approval of manuscript and agreed accountability for all aspects.

## Conflicts of Interest

The authors declare no conflicts of interest.

## Data Availability

The data that support the findings of this study are available from the corresponding author upon reasonable request.
